# A single-center real-life study on the use of medical cannabis in patients with dystonia

**DOI:** 10.3389/fneur.2023.1218300

**Published:** 2023-06-29

**Authors:** Saar Anis, Achinoam Faust-Socher, Diana Sverdlov, Simon Lassman, Neomi Hezi, Omer Anis, Gil Leor, Amos D. Korczyn, Nir Giladi, Tanya Gurevich

**Affiliations:** ^1^Movement Disorder Unit, Neurological Institute, Tel Aviv Sourasky Medical Center, Tel Aviv, Israel; ^2^School of Medicine, Tel Aviv University, Tel Aviv, Israel; ^3^Sagol School of Neuroscience, Tel Aviv University, Tel Aviv, Israel; ^4^St George’s Hospital, University of London, London, United Kingdom; ^5^Department of Urology, Chaim Sheba Medical Center, Ramat-Gan, Israel; ^6^The Mina and Everard Goodman Faculty of Life Sciences, Bar-Ilan University, Ramat-Gan, Israel; ^7^Department of Human Molecular Genetics and Biochemistry, Faculty of Medicine, Tel Aviv University, Tel Aviv, Israel

**Keywords:** medical cannabis, cannabidiol, delta-(9)-tetrahydrocannabinol, dystonia, treatment efficacy, pain

## Abstract

**Background:**

While cannabis-based medicine is being commonly used in patients with movement disorders, there is a scarcity of publications regarding the effect of cannabis on dystonia. We aimed to describe medical cannabis use in patients with dystonia and related pain.

**Methods:**

We employed a structured interview to obtain data on the cannabis treatment regimen, perception of effectiveness and side effect profile. Eligible participants were patients diagnosed with dystonia from the movement disorders unit at the Tel-Aviv Medical Center who had used licensed medical cannabis between January 2019 and January 2021.

**Results:**

Twenty-three subjects were interviewed (11 women, mean age 52.7). The most common way of administration was smoking (*n* = 11). Following an average of 2.5 ± 2.9 years of use, those with widespread dystonia (generalized, hemi and multifocal, *n* = 11) self-reported on a numeric rating scale an average 63% (range 0%–100%) reduction in symptoms of dystonia, while those with more focal dystonia patterns reported a significantly lower treatment effect of 32%. Participants reported a positive impact in related pain and quality of life, with an average rating of 3.8 out of 5 (SD = 1.2, median = 4) and 3.6 out of 5 (SD = 1.15, median = 4), respectively. Most common side effects were dry mouth (65%), sedation (43%), dizziness (39%) and psychiatric disorders (26%). Three patients (13%) discontinued therapy.

**Conclusion:**

A subset of dystonia patients who use medical cannabis under clinical observation reported significant subjective improvement during 30 months of use in average. Further prospective randomized controlled trials are required to examine the effectiveness of cannabis in dystonia.

## 1. Introduction

Dystonia is a hyperkinetic movement disorder that is often accompanied by pain (1). The efficacy of existing medical treatments for dystonia, including anticholinergics, dopamine modulators and other medications, can be variable, contingent upon individual patient factors and the specific subtype of dystonia (2). Suboptimal symptom relief is observed in a subset of patients who do not experience satisfactory outcomes with these conventional treatment modalities. Consequently, there exists a compelling need to investigate alternative therapeutic approaches that may offer potential benefits to individuals who have exhibited inadequate responses to traditional treatments.

Cannabis-based medicine (CBM) is an appealing therapeutic option because cannabinoids, such as Δ9-tetrahydrocannabinol (THC) and cannabidiol (CBD), activate receptors in the endocannabinoid system which is involved in motor coordination and pain modulation. The endocannabinoid receptors are located in several regions of the brain, including the basal ganglia, cortex, hippocampus, and cerebellum, which are all crucial for motor control and pain perception. Therefore, CBM could be a potential therapeutic option for individuals with dystonia who experience pain and motor control difficulties (3, 4).

While the use of CBM has become increasingly popular, conducting robust studies in this area can be challenging due to several factors, including regulatory hurdles, inconsistent formulations, and the utilization of multiple delivery methods. Consequently, the use of CBM remains a controversial issue (5). Three randomized controlled trials have been published with relevance to CBM and dystonia. In a prospective, double-blind, placebo-controlled study conducted in 2022, sublingual administration of medical cannabis (MC) as an oil extract was found to be beneficial for benign essential blepharospasm (6). Two prior studies utilizing orally-administered synthetic THC formulations (nabilone and dronabinol) were unable to demonstrate any significant therapeutic benefit for dystonia symptoms (7, 8). Several systematic reviews conducted in the last decade have reported that cannabinoids may demonstrate efficacy in individual cases or in open label pilot studies. However, the available data is currently insufficient to draw definitive conclusions, and further studies are needed to provide more conclusive evidence (7, 9–14).

The current body of evidence regarding the use of full spectrum MC products is limited to preclinical and clinical studies, and there is a lack of real-world data available. Real-world data, which is collected from patients using MC products in their daily lives, would provide valuable information about the effectiveness and safety of full spectrum MC products in a broader population. Such data could also help identify any adverse effects associated with long-term use, as well as provide insights into the optimal dosing and administration methods for different medical conditions. However, the lack of real-life data may be due to various reasons, including regulatory barriers, limited access to MC products, and social stigma surrounding cannabis use. As the legal and social landscape surrounding cannabis continues to evolve, it is important for researchers to gather more real-world data to inform clinical practice and improve patient outcomes.

In 2013, the Israeli Ministry of Health (MOH) authorized the use of MC for the treatment of dystonia and related pain. The recommended maximum initial dose of 20 g/month, as stated in the Israeli Clinical Guide, is usually administered with a preparation containing 10% THC and 2% CBD (15). The strength of the preparation, whether inflorescence or oil extract, is available in fixed THC:CBD percentage ratios: 0:24, 1:20, 3:15, 5:5, 5:10, 10:2, 10:10, 15:3, and 20:4. Patients’ preferences may help refine the dose, strength, and method of administration. Given almost a decade of experience in treating dystonia with MC, we conducted a real-life analysis with the objective of evaluating the subjective effects of MC on dystonia and related pain, as well as documenting the method of consumption, dosage, and potential side effects. Additionally, we aimed to explore the relationship between treatment regimen and its effects on different types of dystonia.

The strength of our data lies in the fact that MC use is accepted and licensed within our health system, enabling clear delineation of its use among our patients. Our study had both descriptive and investigational aims, with the former being focused on assessing subjective effects and documenting treatment parameters, and the latter aimed at exploring potential differences in treatment response among different dystonia subtypes, way of administration and dosing.

## 2. Methods

### 2.1. Subjects

We conducted a review of electronic medical records from the Movement Disorders Unit (MDU) at the Tel Aviv Sourasky Medical Center (TASMC) for the period between January 1, 2019 and January 1, 2021. The review encompassed patients who were 18 years of age or older, had received a diagnosis of “dystonia” or “blepharospasm” according to the International Classification of Diseases, Ninth Revision (ICD-9), and who went through our pipeline for MC licensure for the purpose of treating dystonia. Patients with significant intellectual disabilities that prevented them from providing consent were excluded from the study.

After obtaining consent from patients, the study investigators (DS, NH or SA) administered the questionnaire either during routine clinic visits or over the phone (with oral consent). The study protocol was approved by our Institutional Review Board.

### 2.2. Questionnaire

The survey assessed various aspects related to dystonia, including demographics, co-existing conditions such as Parkinson’s disease, genetic factors (if applicable), dystonia location, past and present treatment for dystonia, cannabis use duration, method of administration, amount of puffs/drops per single use, monthly dose, and THC:CBD product ratio. The effectiveness of treatment on pain and quality of life (QoL) was evaluated using a five-point Likert-type scale (1 = very dissatisfied, 2 = dissatisfied, 3 = neutral, 4 = satisfied, 5 = very satisfied). Patients were also asked to indicate the percentage of improvement in dystonia severity compared to baseline on a numeric rating scale between 0—no effect to 100—disappearance of the dystonic movements (0%–100%). Furthermore, participants were requested to report whether during use they recall (yes/no) side effects including psychiatric (hallucinations or other psychotic manifestations, anxiety, or depression), cognitive (confusion, memory, visuospatial, or language disturbances), dizziness, sedation, fatigue, dry mouth, gastrointestinal, weight gain, red eyes or other symptoms that were not directly queried. The estimated THC daily dose (ETDD) in grams was calculated using the equation:

$$\mathrm{ETDD}=\frac{\mathrm{Monthly}\;\mathrm{MC}\;\mathrm{dose}\;\left(\mathrm{grams}\right)\;}{30}*\frac{\%\mathrm{THC}\;\mathrm{in cannabis product}}{100}$$

### 2.3. Statistical methods

The data underwent statistical analysis, which involved utilizing descriptive statistics to examine demographic variables such as mean, median, standard deviation (SD), and range. Spearman’s rank correlation was used for nonparametric variables, while Pearson’s correlation was used for parametric variables to perform correlation analysis. The two-sided student’s *t*-test and two-sided Mann Whitney test were used to estimate differences between means of parametric and non-parametric variables, respectively.

Furthermore, a linear regression model was employed to test the relationship between mean improvement percentage of dystonia and THC dose (grams/month), MC formulation monthly dose (grams) and ETDD. The statistical significance level for both parametric and nonparametric testing was set at 0.05. The data analysis was carried out using the R language and environment for statistical computing (16), and Graph-Pad Prism version 9.0 was used to generate graphs. A biomedical statistician, GL, performed all statistical analyses.

## 3. Results

### 3.1. Demographics and disease characteristics of our cohort

Among the overall collective of patients diagnosed with dystonia who were being followed up in the MDU at TASMC during the study period (*n* = 125), we identified 36 patients (29%) whom their treating physician had recommended the use of MC. Of these patients, five (14%) had previously used MC for dystonia but had discontinued treatment before the study period. Three patients refused to participate, two had obtained their MC license from the MOH but had not yet initiated MC treatment at the time of recruitment, and three were still in the process of obtaining their MC license. Ultimately, a total of 23 patients consented to participate in the study and were interviewed either by telephone or during a follow-up visit. The study cohort consisted of 11 women and 12 men, and a mean age of 52.7 years. The dystonia phenotype in the study participants was distributed as follow: six patients had focal dystonia, five with segmental dystonia, one with multifocal dystonia, two with hemi-dystonia and nine with generalized dystonia. Dystonia was determined to be idiopathic in 13 patients, while in four patients, the etiology of dystonia was linked to genetic mutation, with two patients presenting DYT1 mutation and the other two presenting DYT6 mutation. Six patients had dystonia as part of their Parkinson’s disease, either secondary to levodopa treatment or as a result of the disease itself. Among the study participants, 14 individuals were currently receiving medications for dystonia in addition to MC: baclofen (*n* = 5), anticholinergics (*n* = 3), benzodiazepines (clonazepam, *n* = 6), levodopa/carbidopa (*n* = 1), botulinum toxin injections (*n* = 1), and topiramate (*n* = 1). Notably, 74% of the patients had previously undergone a trial with at least one therapy for dystonia.

### 3.2. MC consumption habits

The majority of our study participants (47.8%) consumed MC through inhalation, either by smoking or vaporization. The average duration of MC use was 2.5 years, with an average frequency of consumption of 3.3 times per day. The average THC percentage of the MC products used was 10%. The mean monthly dose of MC consumed was 22.3 grams, and the ETDD was approximately 100 mg. Study cohort characteristics and details of MC consumption habits are summarized in Table 1.

**Table 1:** Study cohort characteristics and MC consumption habits.

Age (years)	52.7 (18.1)
Gender f/m	11/12
Age of dystonia onset (years)	37.4 (22.5)
Disease duration (years)	15.26 (11.5)
Mode of consumption (oil/inhalation/both)	10/11/2
Cannabis duration of use (years)	2.5 (2.9)
Current monthly dose (grams)	22.3 (20.1)
Percent THC in MC product	10.6 (6.6)
Percent CBD in MC product	8.0 (5.6)
Frequency of consumption (times per day)	3.3 (4.3)
Quantity of puffs/drops in each use	7.0 (5.6)
Estimated THC daily dose (mg)	101.4 (150.7)
Estimated CBD daily dose (mg)	51.4 (41.6)
Percentage of dystonia improvement (0%–100%)^a^	47.5 (35.4)

Data are presented as mean (standard deviation). f/m, female/male; mg, milligrams; MC, medical cannabis; THC, tetrahydrocannabinol; CBD, cannabidiol.

a *n* = 22, one patient resigned to answer.

### 3.3. MC reported efficacy

**Figure 1:**
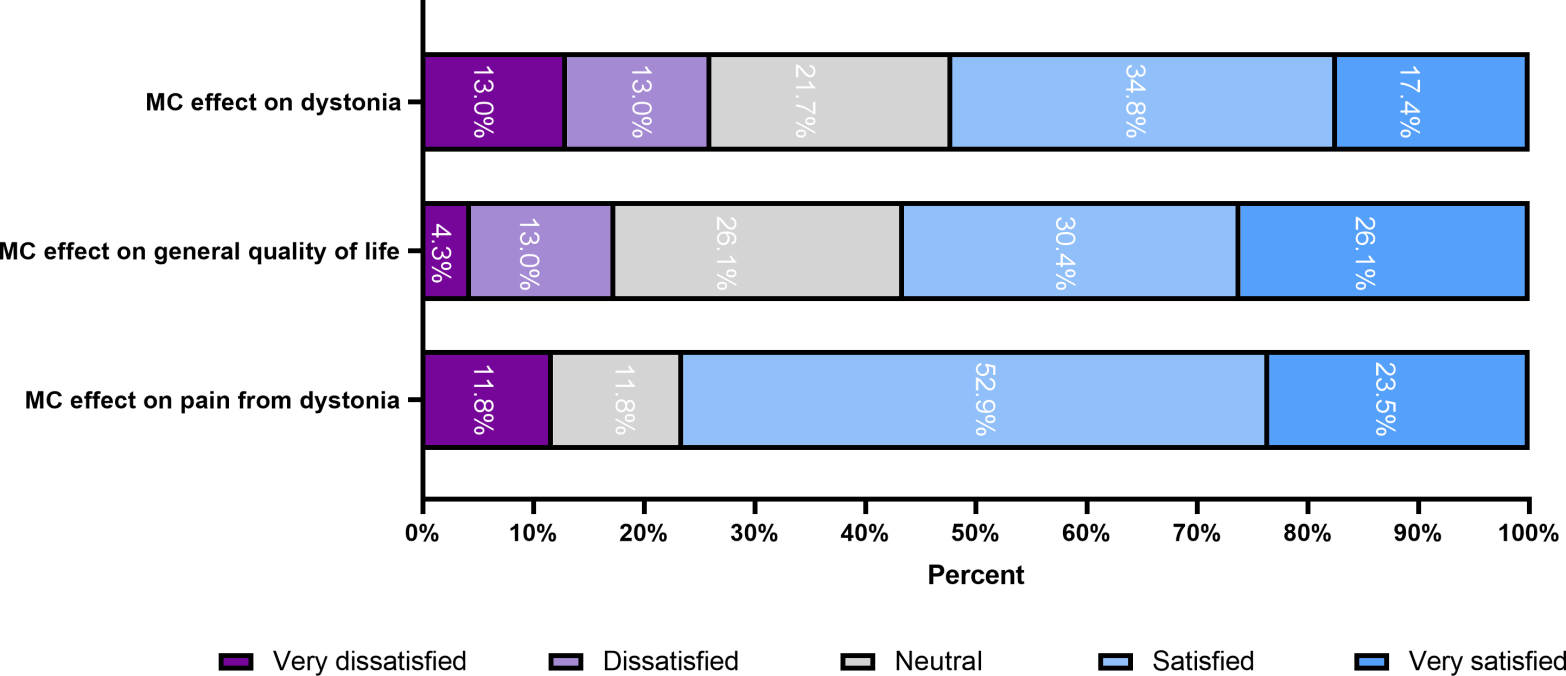
MC effect on dystonia, related pain and QoL using a five-point Likert-type scale. MC, Medical cannabis; QoL, quality of life.

The mean self-reported response of dystonia during MC treatment on a Likert scale was 3.3 out of 5 (SD = 1.29, median = 4), suggests that the majority of participants expressed a positive response to MC treatment for dystonia. The response for related pain and QoL was also favorable, with 3.8 out of 5 (SD = 1.2, median = 4) for pain, and *t* 3.6 out of 5 (SD = 1.15, median = 4) for QoL. These outcomes are depicted in Figure 1.

**Figure 2:**
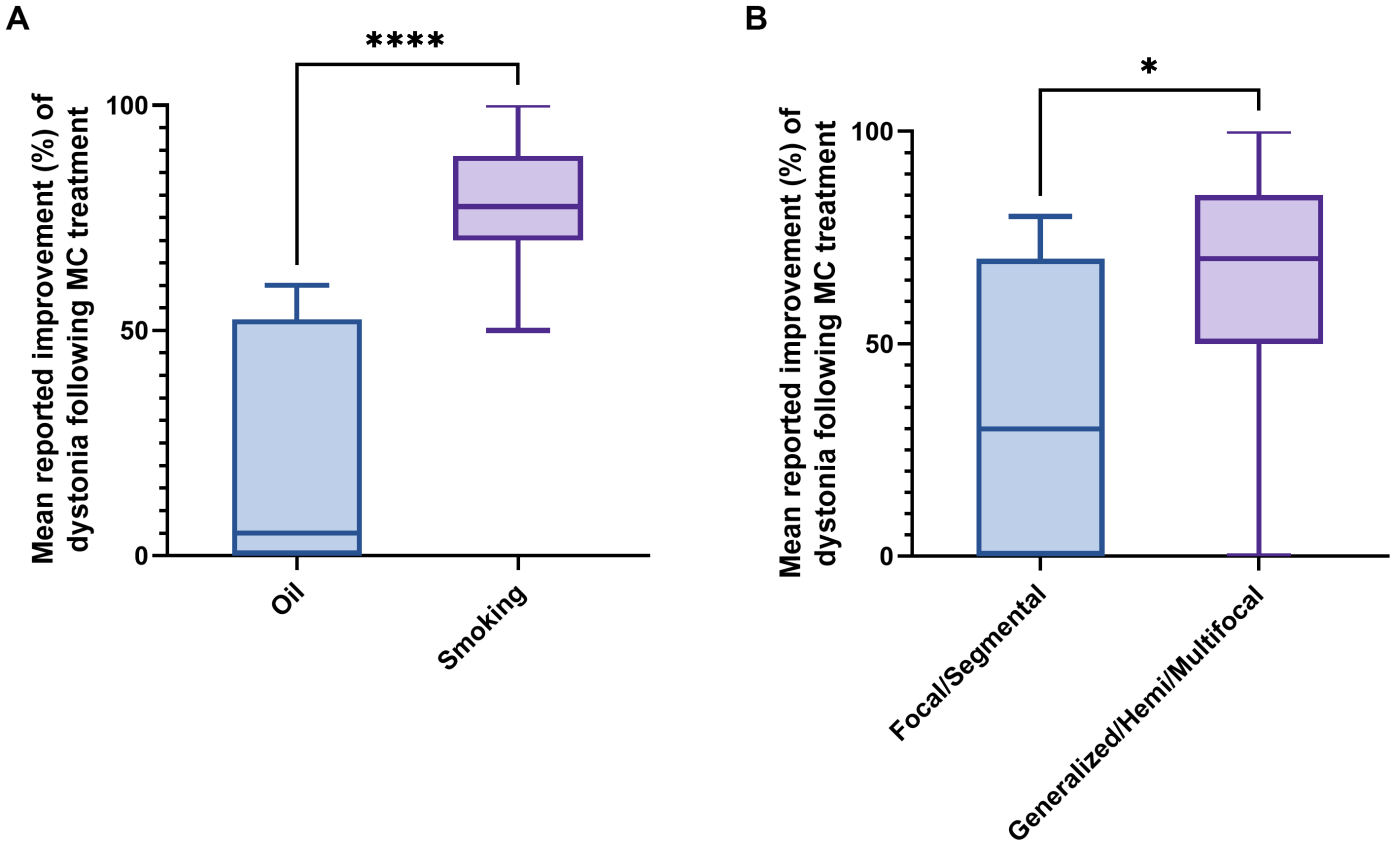
Effect of dystonia type and method of MC administration on subjective improvement in dystonia. **(A)** Effect of cannabis method of administration on subjective treatment efficacy for dystonia. **(B)** Effect of dystonia type on subjective treatment efficacy for dystonia. MC, Medical cannabis. * *p* -value = 0.04, ****p-value = 0.0003.

To evaluate the effect of the form of MC administration on subjective improvement in patients, a two-sided Mann–Whitney test was performed. Results indicated that inhaled MC was associated with greater subjective improvement in dystonia compared to sublingual MC oil (*p*-value = 0.00033). The mean percentage improvement was 78.5% for smoking compared to 21% for sublingual oil (Figure 2A). Patients with widespread dystonia (generalized, hemi, and multifocal dystonia, *n* = 11) reported higher benefit (63% vs. 32% on average, *p* = 0.04) compared to patients with focal or segmental dystonia (*n* = 11), as demonstrated in Figure 2B.

**Figure 3:**
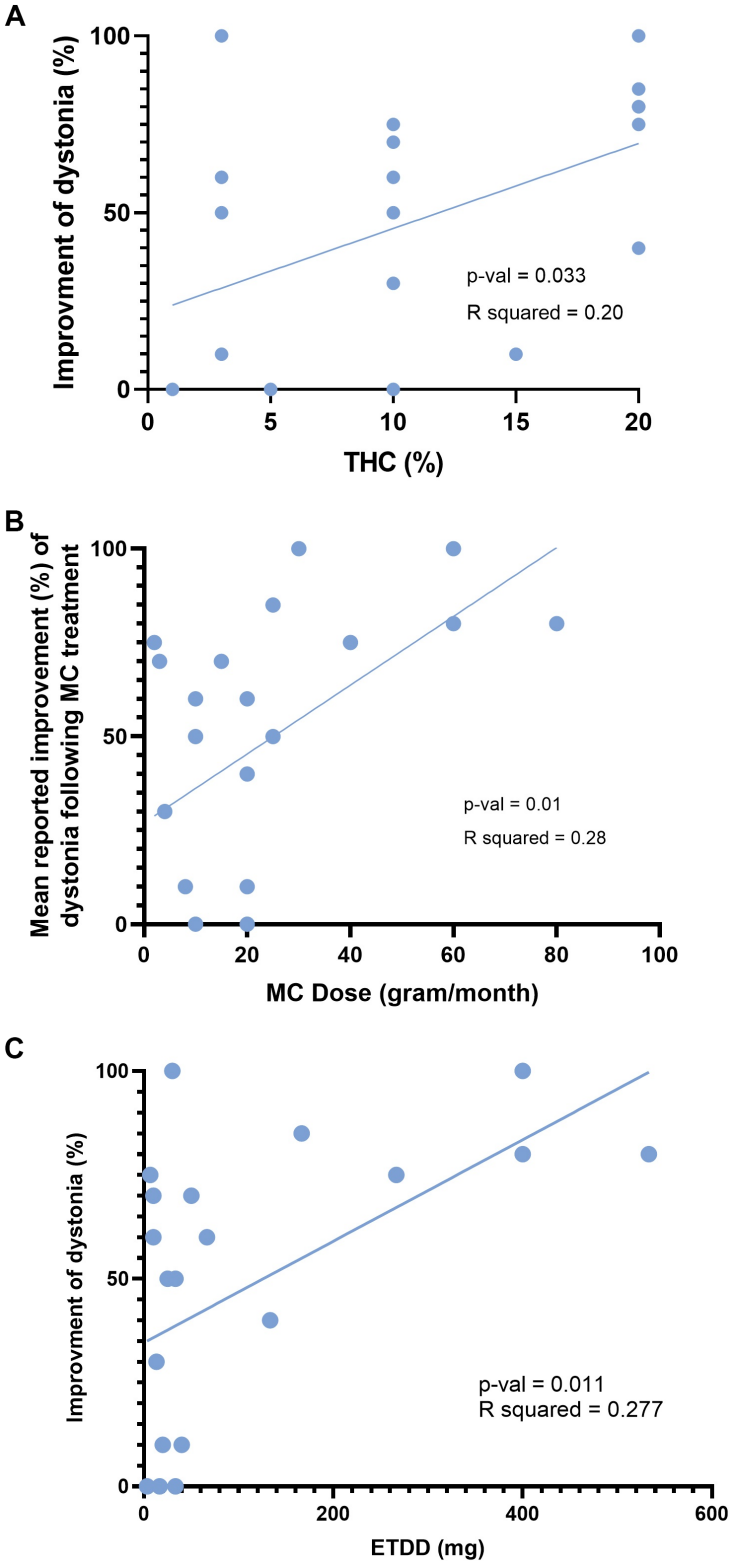
Influence of MC dose and THC content on improvement of dystonia. This figure depicts the correlation between reported improvement (%) of dystonia and three variables: **(A)** THC percentage within the cannabis product. **(B)** Total monthly consumption of medical cannabis in grams. **(C)** Estimated THC daily dose in milligrams. MC, Medical cannabis; THC, Δ9-tetrahydrocannabinol; ETDD, estimated THC daily dose.

A linear regression model was employed to investigate the relationship between improvement in dystonia and parameters related to MC treatment. The analysis revealed a positive correlation between improvement in dystonia and three variables: (1) THC percentage within the MC product (*p* = 0.03, *R* squared = 0.2), as illustrated in Figure 3A; (2) MC monthly dose in grams (*p* = 0.01, *R* squared = 0.28), as depicted in Figure 3B; and (3) ETDD, a variable that combines THC product percentage and MC monthly dose (*p* = 0.01, *R* squared = 0.27), as demonstrated in Figure 3C.

### 3.4. MC reported side effects and treatment termination

Dry mouth (65%), sedation (43%), and dizziness (39%) were the most common side effects reported by patients. Twenty-five percent of patients (*n* = 6) experienced psychiatric side effects, with anxiety reported in three cases (one of which included hallucinations), and worsening mood reported in three cases (one of which included suicidal ideation) after the initiation of treatment. Treatment modifications (such as administering products with lower THC percentage) resolved side effects in five patients, while one patient had to discontinue treatment entirely. Two patients ceased treatment due to perceived inefficacy. Of the three patients who discontinued treatment (13%), two did so within a few weeks of initiation, and one after 2 years.

## 4. Discussion

Our real-life observational single-center study suggests that MC may provide benefits for some patients with dystonia, particularly those with more widespread or generalized forms of the condition. However, it is important to note that there were several reported side effects associated with MC use, including dry mouth, sedation, dizziness, and psychiatric side effects in some patients. These side effects were manageable in most cases, but did lead to treatment modification in some patients and complete cessation in one patient. It is important to consider the possible mechanisms by which MC might alleviate dystonia. One potential explanation is that the endocannabinoid system, which is abundant in brain areas related to movement control and dystonia such as the basal ganglia and cerebellum, could be involved in the lack of inhibition that leads to excessive muscle contraction in dystonia (17, 18). For example, the CB1 receptor is expressed in GABAergic medium spiny neurons in the striatum and substantia nigra, as well as in glutamatergic cortex projections, all of which play a role in dystonia pathophysiology (19, 20). THC and other cannabinoids have been shown to enhance GABA release and reduce its reuptake while also reducing glutamatergic flow, which could improve movement inhibition (21), as has been suggested in other hyperkinetic movement disorders (22, 23).

Based on our collected data, we sought to identify factors that may impact the perceived improvement in dystonia. Our findings suggest that the spread of dystonia may be a relevant factor to consider. Specifically, patients with focal or segmental dystonia (comprising half of our cohort) reported significantly less benefit compared to those with more widespread dystonia. This observation supports the notion that medication efficacy may vary depending on the type of dystonia. For example, anticholinergics are generally more effective for generalized or segmental dystonias (24, 25). These differences may be related to specific biochemical or physiological mechanisms underlying the pathogenesis of dystonia that should be further investigated. It is important to note, however, that medication efficacy is likely multifactorial and may vary on an individual basis.

Additionally, we observed that the method of administration appeared to be a factor in the perceived improvement of dystonia. Patients who inhaled dried buds reported the greatest improvement, while half of the patients who consumed oil extract reported no improvement in symptoms. Interestingly, most studies on cannabis-based therapies for dystonia have focused on oral consumption (9), with only a few case reports describing the benefit of inhaled MC (26, 27). The difference in reported benefit may be attributed to the pharmacokinetics of cannabinoids. Inhaled THC reaches peak plasma concentration within 8–12 min, while oil extract takes 30 min to reach the first measurable plasma levels and peaks at 3–4 h (28). Furthermore, smoking THC achieves a higher plasma concentration [*C*(max), (microgram/L) (6)] compared to sublingual oil (29). Nevertheless, it is essential to consider the risks associated with smoking, such as respiratory and vascular complications, when prescribing inhaled cannabis. Overall, our findings suggest that the method of administration should be carefully considered to balance potential benefits with risks.

Our study found that total monthly consumption of MC, THC percentage within the product, and ETDD were positively correlated with perceived benefit in dystonia, indicating a potential role for THC in its treatment. This findings aligns with the increasing use of synthetic THC in other hyperkinetic movement disorders such as tics in Tourette syndrome (30) and Huntington’s disease (31), which has been previously investigated in small data sets. Moreover, previous investigations have also suggested a possible benefit of THC in the treatment of dystonia, which our results support (8, 32).

This study has several limitations that should be considered. The sample size was small and limited by selection bias, as we did not collect data from patients who did not agree to participate. Additionally, all results were based on self-reported measures without objective confirmation and were collected retrospectively at a single point in time without regular follow-up or randomization, which may be subject to false memories and reporting. The retrospective design of this study did not allow for the determination of the time course of the positive response to treatment, nor did it enable the assessment of indirect measures of efficacy, such as a decrease in the use of other drugs. Furthermore, we were unable to elucidate the temporal profile of the response to MC treatment in patients with dystonia, including whether there is a development of tolerance or improvement in the response over the course of MC treatment. Finally, our cohort was diverse, including patients with different etiologies and spreads of dystonia, and using various MC strains with different THC:CBD compositions and from different growers, which may have influenced the results.

To strengthen the evidence for CBM in dystonia, future research should include well-designed randomized double-blind placebo-controlled trials with larger sample sizes to minimize bias and consider placebo effects. Validated motor assessment scales, such as the Burke–Fahn–Marsden dystonia rating scale or the global dystonia severity rating scale, should be used to measure motor symptoms. Specific measures should be incorporated to assess non-motor responses like pain, quality of life, sleep disturbances, and psychiatric symptoms. Additionally, participants should be categorized based on dystonia subtype and etiology to determine if certain subtypes benefit more from CBM treatment. Developing standardized protocols for CBM administration, including dosing regimens, formulations, and administration routes (e.g., inhalation, oral, or sublingual), will enhance consistency and result comparisons. Lastly, comprehensive data collection on adverse events, covering both motor and non-motor side effects, is crucial to understand the safety profile of CBM in dystonia. Implementing these recommendations will establish a strong evidence base for CBM in dystonia, supporting the development of personalized treatment approaches.

In conclusion, our findings indicate that THC-containing MC products may be a promising starting point for further research into the therapeutic benefits of CBM for dystonia in patients with widespread symptoms. However, more randomized, placebo-controlled studies are necessary to determine the most effective THC dose, with or without the addition of CBD and other cannabinoids.

## Data availability statement

The raw data supporting the conclusions of this article will be made available by the authors, without undue reservation.

## Ethics statement

The studies involving human participants were reviewed and approved by Helsinki Committee (IRB) Tel Aviv Medical Center. Written informed consent for participation was not required for this study in accordance with the national legislation and the institutional requirements.

## Author contributions

SA, AF-S, SL, AK, NG, and TG played a key role in the conception and design of the study. SA, DS, and NH interviewed the patients and provided valuable clinical insights. SA, OA, and GL contributed to statistical analysis. OA provided valuable information in the field of medical cannabis. SA wrote the initial draft of the manuscript, while SL, AF-S, OA, AK, NG, and TG contributed to various sections of the manuscript. All authors contributed to the article and approved the submitted version.

## Conflict of interest

The authors declare that the research was conducted in the absence of any commercial or financial relationships that could be construed as a potential conflict of interest.

## Publisher’s note

All claims expressed in this article are solely those of the authors and do not necessarily represent those of their affiliated organizations, or those of the publisher, the editors and the reviewers. Any product that may be evaluated in this article, or claim that may be made by its manufacturer, is not guaranteed or endorsed by the publisher.
